# Random Knotting in Fractal Ring Polymers

**DOI:** 10.1021/acs.macromol.2c01676

**Published:** 2022-09-08

**Authors:** Phillip M. Rauscher, Juan J. de Pablo

**Affiliations:** † Pritzker School of Molecular Engineering, University of Chicago, Chicago, Illinois 60637, United States; ‡ Materials Science Division (MSD) and Center for Molecular Engineering (CME), Argonne National Laboratory, Lemont, Illinois 60439, United States

## Abstract

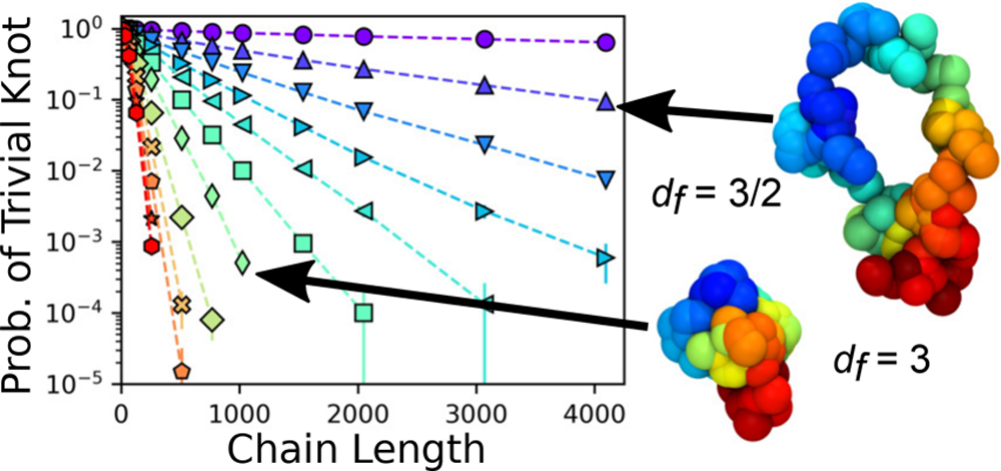

Many ring polymer systems of physical and biological interest exhibit both pronounced topological effects and nontrivial self-similarity, but the relationship between these two phenomena has not yet been clearly established. Here, we use theory and simulation to formulate such a connection by studying a fundamental topological property—the random knotting probability—for ring polymers with varying fractal dimension, *d*_*f*_. Using straightforward scaling arguments, we generalize a classic mathematical result, showing that the probability of a trivial knot decays exponentially with chain size, *N*, for all fractal dimensions: *P*_0_(*N*) ∝ exp(−*N*/*N*_0_). However, no such simple considerations can account for the dependence of the knotting length, *N*_0_, on *d*_*f*_, necessitating a more involved analytical calculation. This analysis reveals a complicated double-exponential dependence, which is well supported by numerical data. By contrast, functional forms typical of simple scaling theories fail to adequately describe the observations. These findings are equally valid for two-dimensional ring polymer systems, where “knotting” is defined as the intersection of any two segments.

## 1 Introduction

In recent years, ring polymers have become one of the most intensely studied subjects of soft matter physics research, as these molecules exhibit fascinating topological interactions.^1−4^ Such interactions lead to remarkable dynamical and rheological behavior,^5−7^ play a fundamental role in mechanically interlocking polymers and molecular machines,^8,9^ and have close connections with the physics of DNA and cellular chromatin.^10−15^ The latter example has also motivated research on the structure and dynamics of polymers with varying fractal dimension, *d*_*f*_,^16−21^ as chromatin in the nucleus appears to have 2.5 ≤ *d*_*f*_ ≤ 3 depending on the length scale,^22−24^ differing from the more familiar values of *d*_*f*_ = 2 for the ideal chain and *d*_*f*_ = 3 for the fractal globule.^25^ These variations in fractal dimension are not merely academic: they are associated with gene expression and have implications in the diagnosis and prognosis of various medical conditions.^26−29^ Clearly, the interplay between topology and fractal dimension is a topic of great significance in chromatin biophysics and beyond.

In light of the importance, researchers have recently begun to examine the combined effects of topology and self-similarity,^30,31^ but many of the most basic questions remain unanswered or simply unasked. For example, among the most fundamental problems of polymer topology is determining the probability that a closed curve of *N* segments will form a nontrivial knot, denoted *P*(*N*).^1,2^ Although this topic has received a great deal of attention for ideal and self-avoiding polymers, the manner in which *P*(*N*) depends on *d*_*f*_ is entirely unknown. One expects *d*_*f*_ to have a considerable effect on the topological properties of ring polymers because the molecule size scales as ; rings with larger fractal dimension should be effectively denser, making segment contacts and crossings more likely. However, a more quantitative understanding is clearly needed for studying the complex systems highlighted above. Here, we offer new insights into this problem using theory and numerical experiments. We first apply scaling arguments to obtain the functional form of *P*(*N*), finding that the classic mathematical result 1 – *P*(*N*) = *P*_0_(*N*) ∝ exp(−*N*/*N*_0_) holds for arbitrary *d*_*f*_, which is verified through numerical calculations. We then perform a detailed statistical calculation to determine the relationship between the random knotting length, *N*_0_, and *d*_*f*_. The result is a complex double-exponential form that cannot be rationalized by typical scaling theories. We verify these results numerically and show that they apply also to lower dimensional analogues.

## 2 Model and Methods

### 2.1 Theoretical Model

For concreteness, we focus on a simple physical model to develop our theory and evaluate it numerically: the so-called “beta model”,^16,20^ which we adapt to ring polymers. The conformation of the polymer is written in terms of the normal/Fourier modes:
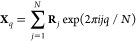
1where **R**_*j*_ is the position of the *j*th repeat unit (or bead). These modes represent structure on the scale of *N*/2*q*′ segments, where *q*′ = min(*q*, *N* – *q*). The mode *q* = 0 corresponds to the center of mass, which is irrelevant for our discussion and ignored hereafter. An effective Hamiltonian is written as
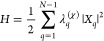
2so that the modes are Gaussian distributed with zero mean and variance 1/λ_*q*_^(χ)^. The eigenvalues are given by
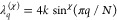
3where *q* = 0, 1, ..., *N* – 1 and *k* = *dk*_B_*T*/*b*^2^ is the spring constant for an isolated dumbbell in *d* dimensions.^a^ We use units such that *k*_B_*T* = *b* = 1, so that the model is effectively athermal. For a fractal polymer, the mean-squared mode amplitudes scale as ;^32,33^ choosing χ = 1 + 2/*d*_*f*_ therefore reproduces the desired structure at large length scales, as can be verified by the Taylor expansion of λ_*q*_^(χ)^. For *d*_*f*_ = 2, the ordinary ideal bead–spring (Rouse) model is recovered.

### 2.2 Computational Methods

To test our theoretical predictions, we perform numerical experiments using the model described above. Polymer configurations with desired *d*_*f*_ are sampled by generating *N* – 1 Gaussian random vectors with variances of 1/λ_*q*_^(χ)^ for *q* ≥ 1; the bead positions then follow via the inverse Fourier transform.^34^ The knot type for each configuration is determined by calculating the Alexander polynomial^35^ using the Topoly^36^ and pyknotid^37^ packages. For each set of parameters (*d*_*f*_, *N*), we collect between 2 × 10^4^ and 10^5^ sample configurations.

## 3 Results and Discussion

### 3.1 Form of the Knotting Probability

#### 3.1.1 Scaling Arguments

To begin, we consider the functional form of *P*(*N*). The famous Frisch–Wasserman–Delbrück conjecture,^38,39^ formulated in the early 1960s, argues that this probability should approach unity as *N* increases. This was later proven mathematically for several polymer models,^40−44^ both ideal and self-avoiding, and on- and off-lattice. In particular, it was shown that the knotting probability approaches unity with an exponential form
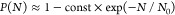
4where the random knotting length, *N*_0_, depends on the details of the physical model under consideration. Numerical simulations have also convincingly demonstrated the validity of eq 4 for a variety of polymer systems,^45−51^ suggesting a highly universal relationship. This naturally leads to the question: does this form hold for arbitrary *d*_*f*_? This query can be addressed at the scaling level by extending the exposition originally offered by Grosberg.^52^

The polymer can be coarse-grained into blobs of *g* segments. If the polymer is knotted, the knot can be manifested in two different ways. First, the coarse-grained chain of *N*/*g* blobs may be knotted; because the polymer is self-similar, the associated probability is simply *P*(*N*/*g*). Second, the knots may exist within the blobs. We express the probability that a blob of *g* segments is knotted by the function ϕ(*g*).^b^ Assuming the blobs are independent, we can now write an equation for *P*(*N*):
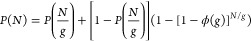
5The first term on the right accounts for knotting at the global scale, and the second accounts for the probability of finding a knot in at least one of the *N*/*g* blobs. One may also consider knots that form due to entanglements at the interface between contacting blobs. However, a mean-field treatment suggests that such considerations merely introduce a correction that may be ignored in the limit *N* → *∞* for physically relevant fractal dimensions (see Appendix A). Equation 5 may be rearranged to yield an expression for ϕ(*g*):
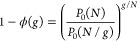
6where *P*_0_(*N*) = 1 – *P*(*N*) is the probability that the polymer is *un*knotted. Because the left-hand side is independent of *N*, all *N*-dependence on the right must vanish. This is only possible if the function *P*_0_(*N*) has an exponential character, i.e., *P*_0_(*N*) = const × exp(−*N*/*N*_0_), consistent with the rigorous mathematical result mentioned earlier. Inserting this form into eq 6, we find ϕ(*g*) = 1 – exp[−(*g* – 1)/*N*_0_]. Because *N*_0_ is typically on the order of 10^2^ or more,^53^ we have the approximate equality ϕ_0_(*N*) ≈ const × *P*_0_(*N*), where ϕ_0_(*N*) = 1 – ϕ(*N*). Note that we have specified neither the length scale for the coarse-graining, *g*, nor the fractal dimension, *d*_*f*_. Thus, we see that self-similarity *itself* implies the exponential form of the knotting probability. The effects of *d*_*f*_ (as well as the specifics of the physical model) enter only through constants such as the knotting length, *N*_0_.

#### 3.1.2 Numerical Results

**Figure 1:**
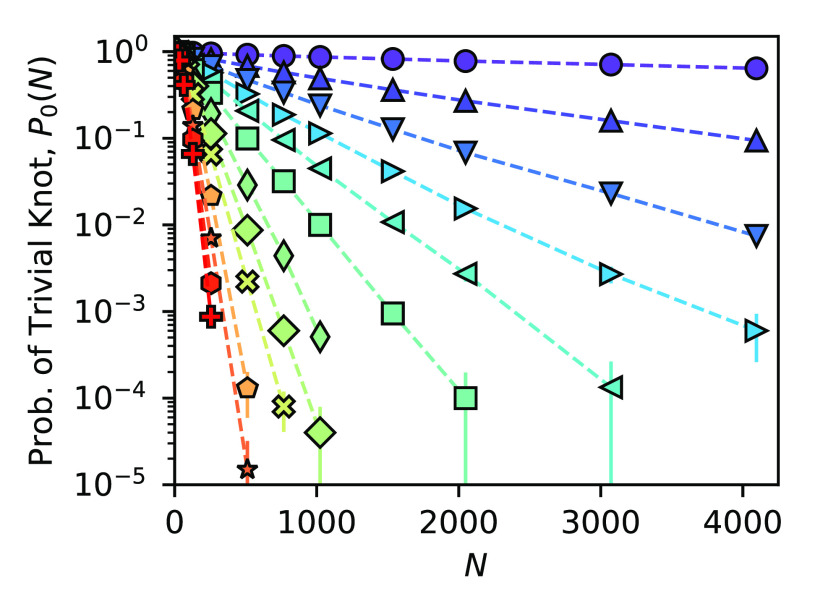
Probability of finding a trivial knot (0_1_) as a function of *N* for ring polymers with fractal dimension *d*_*f*_ = 6/5 (circles), 3/2 (upward triangles), 5/3 (downward triangles), 9/5 (right triangles), 2 (left triangles), 11/5 (squares), 5/2 (diamonds), 3 (wide diamonds), 7/2 (×’s), 4 (pentagons), 9/2 (stars), and 5 (hexagons). The decay is exponential for all systems.

The probabilities of the trivial knot for ring polymers with 6/5 ≤ *d*_*f*_ ≤ 5 are shown in Figure 1 on a logarithmic scale. The clear linear dependence of ln *P*_0_(*N*) on *N* for all *d*_*f*_ indicates that all systems exhibit the exponential behavior predicted above. On the basis of these results, we conclude that the exponential form of the knotting probability is valid for all fractal dimensions, although the knotting lengths *N*_0_ (i.e., the slopes) clearly have a strong dependence on *d*_*f*_. Note that although the finite compressibility of real polymers prohibits fractal dimensions greater than the spatial dimension for long chains, we have nevertheless included some values above this threshold because they sometimes appear in polymer models of physical interest^54,55^ and because they serve to demonstrate the generality of our results.

### 3.2 Random Knotting Length and Fractal Dimension

#### 3.2.1 Conceptual Arguments and Limiting Cases

Next, we aim to understand and quantify the dependence of *N*_0_ on *d*_*f*_. Intuitively, this should be a strictly decreasing function: as *d*_*f*_ grows, the polymers occupy less volume and segments far apart along the chain contour are more likely to overlap, leading to more opportunity for knotting. In the limit of large *d*_*f*_, *N*_0_ should approach a small but finite value, as there is a minimum required number of segments needed for knotting (known as the “stick number”, equal to six for the simplest knot, 3_1_^35^). Of course, for such small values of *N*, the chain is not fractal in any meaningful sense. Nevertheless, the existence of a rigorous mathematical lower bound for knotting implies that *N*_0_ is similarly bounded.

At the other limit, *d*_*f*_ → 1, the situation is more subtle. In particular, the ring closure requirement implies that such small fractal dimensions are only realized quasi-locally, and the “global” fractal dimension is always greater than unity. Even at this local level, the knotting cannot be achieved with *d*_*f*_ = 1 since knotting requires that the chain “double back” on itself, which is not possible for rigid rod conformations. Thus, we may observe a divergence of *N*_0_ as *d*_*f*_ → 1 but do not entertain any particular expectations.

Between the limits discussed above, the form of the dependence is unknown and difficult to anticipate on the basis of scaling arguments. For example, postulating that knotting becomes probable once the local segment density becomes larger than some critical value would lead to a function with some residual *N* dependence, which is at odds with the simple exponential behavior demonstrated for all *d*_*f*_, as described above. Moreover, scaling results for confined knotted systems cannot be applied here since those systems include walls that help randomize segmental orientations,^56^ whereas fractal polymers can possess long-ranged correlations.

#### 3.2.2 Statistical Calculation

Lacking a simple scaling argument, we carry out a more direct calculation on the basis of statistical mechanics. For concreteness, we use the same model described above, although the results are generally applicable to any model so long as variations in large-scale fractal dimension may be introduced without significantly altering the local polymer conformations (see Discussion section below). The (configurational) partition function of the system is written . We introduce also the *constrained* partition function,  where the symbol *∫*_0_ indicates that one only carries out the integration over regions of phase space for which the polymer is unknotted. The probability of observing a trivial knot may then be written *P*_0_(*N*) = *Z*_0_/*Z*. Taking the derivative with respect to χ, we have
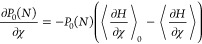
7where the angled brackets ⟨...⟩_0_ and ⟨...⟩ denote ensemble averages in the constrained (unknotted) and unconstrained ensembles, respectively. We now use eq 4 to evaluate the derivative on the left-hand side of the previous formula and use eqs 2 and 3 to evaluate the derivatives on the right. After applying the equipartition theorem, , for the unconstrained average, we finally obtain
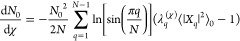
8Further progress requires that we understand how the polymer structure is affected by knotting (or unknotting), which involves some approximations on account of the mathematical difficulty; this is discussed in the following.

To determine the values of , we make use of the classic arguments of Grosberg.^52^ In short, chain segments with more than *N*_0_ monomers repel one another through a topological excluded volume. As a result, the polymer conformations are not affected on small length scales but become self-avoiding on larger ones. Given the self-similar nature of the polymers and the physical interpretation of the modes **X**_*q*_ as describing the polymer structure on the scale of *N*/2*q*′ segments, we postulate the following relations:
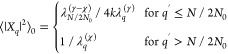
9where γ is a new scaling exponent reflecting the self-avoiding nature of the polymer at large length scales. The factor of  in the first line of eq 9 ensures that the mode amplitudes are continuous at *q*′ = *N*/2*N*_0_. Clearly, we must have γ ≥ χ because excluded volume interactions can only swell the chain.

Next, we require an estimate for γ, which we obtain using the generalized Flory argument proposed by Matsushita et al.^57^ in the context of fractional Brownian motion, which is also associated with fractal polymer systems.^30,58^ The free energy of a self-avoiding fractal polymer is expressed as the sum of contributions from an interacting gas of segments and an ideal Gaussian chain with a given fractal dimension, *d*_*f*_:
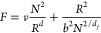
10Here, *v* is the excluded volume parameter. Minimizing this free energy with respect to *R* leads to
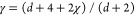
11Despite the well-known shortcomings of Flory-type arguments,^59^ this relation has been shown to be quite accurate in a variety of systems^31,60,61^ and also agrees well with our own numerical data (see Figure S1) and so may be used with a degree of confidence. Moreover, the key analytical results of this study do not actually depend on the precise value of the exponent γ, so even if the exact dependence on *d*_*f*_ were unknown, the main points of the paper would remain valid (*vide infra*). On the other hand, eq 11 implies γ = χ for *d*_*f*_ = 3/2; that is, the topological constraints no longer affect the chain conformations, which is not physically plausible as (effective) excluded volume interactions must swell the chain as mentioned earlier. In general, we do not expect eq 11 to remain valid for small fractal dimensions, in particular *d*_*f*_ ≤ 1.7, corresponding to a self-avoiding ring.

Upon combining eqs 8 and 9, the modes with *q*′ > *N*/2*N*_0_ drop out of the sum as these correspond to short length-scale structure, which is unaffected by the topological constraint. Moreover, because of the degeneracy in λ_*q*_^(χ)^ (due to the periodic nature of the sine function), we can consider only terms for which *q* ≤ *N*/2*N*_0_ (note the lack of an apostrophe!) and multiply the results by 2. The differential equation now reads
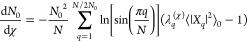
12Because *N*_0_ is usually fairly large (on the order of 10^2^ even for very large *d*_*f*_), all terms in eq 12 have *q*/*N* ≪ 1. We therefore expand the quantities λ_*q*_^(χ)^ and sin(π*q*/*N*) about *q*/*N* = 0 and ignore terms of order (*q*/*N*)^2^ or higher. In both cases, we retain only a single term and have λ_*q*_^(χ)^ ≈ 4*k*(π*q*/*N*)^χ^ and sin(π*q*/*N*) ≈ π*q*/*N*. With these substitutions and eq 9, we have
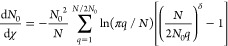
13where δ = γ – χ. We now express the logarithmic factor in eq 13 as , which allows us to separate the sum:
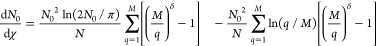
14where we have defined *M* = *N*/2*N*_0_. This representation makes explicit the dependence on ln *N*_0_, which is seen in the first term on the right. In the limit *N* → *∞*, *M* also becomes very large, and we may approximate the sums as integrals:
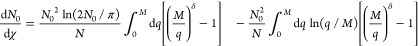
15It is worth noting that the sums in eq 14 can in fact be evaluated exactly in terms of Riemann and Hurwitz zeta functions. However, after taking the limit *N* → *∞*, one arrives at the same results, so we prefer to present the simpler integral method instead. The integrations are now performed to obtain
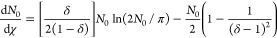
16Collecting terms proportional to *N*_0_ ln *N*_0_ and *N*_0_, dividing both sides by *N*_0_, and using the definition μ = ln *N*_0_, we have
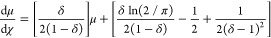
17Finally, we use the definition of χ = 1 + 2/*d*_*f*_ to change variables and substitute eq 11 for γ in three dimensions to arrive at
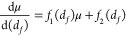
18where *f*_1_(*x*) and *f*_2_(*x*) are certain algebraic functions:
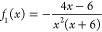
19
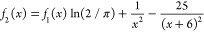
20Although these functions depend on the details of the polymer model in question, the general form of eq 18 is determined only by the scaling relations eqs 2, 3, and 9, which should be valid for any large fractal ring polymer, regardless of the “molecular” details. For most of the values of *d*_*f*_ considered here, *f*_1_(*d*_*f*_) and *f*_2_(*d*_*f*_) are roughly constant (see Figure S3), which leads to an approximate solution:
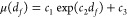
21The form of eq 21 is noteworthy: it indicates that the knotting length *N*_0_(*d*_*f*_) has a double-exponential character. Accordingly, the knotting probability is a *triple* exponential in *d*_*f*_. It is not clear what kinds of scaling arguments (if any) could explain this nontrivial dependence. However, for relevant choices of the constants *c*_*i*_ (see below), eq 21 satisfies the anticipated attributes of *N*_0_(*d*_*f*_), i.e., strictly decreasing and with a finite limit at *d*_*f*_ → *∞*.

#### 3.2.3 Numerical Validation

**Figure 2:**
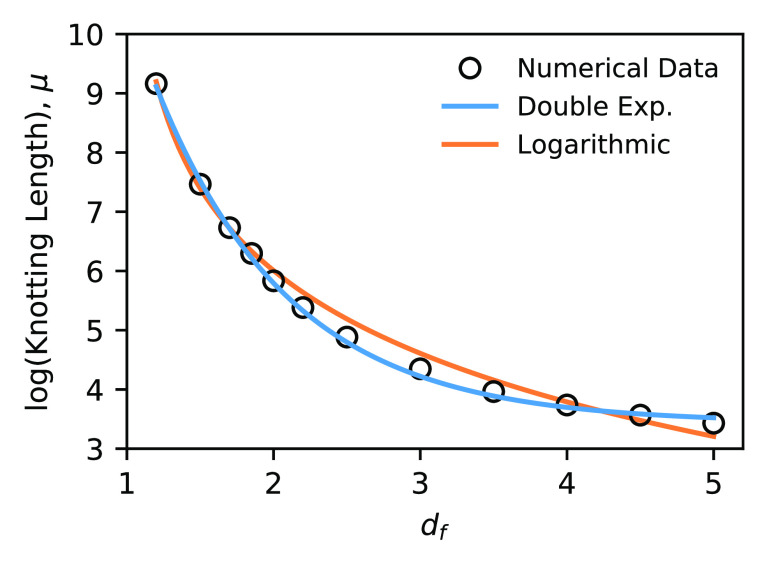
Logarithm of the knotting length ln *N*_0_ ≡ μ as a function of *d*_*f*_. Statistical errors are much smaller than the size of the markers. The data are well fit by the double-exponential function, eq 21, but not by a logarithmic form, μ = *c*_1_ ln(*d*_*f*_ + *c*_2_) + *c*_3_.

To verify the double-exponential character, we fit the data sets in Figure 1 according to eq 4 and plot the resulting values of μ = ln *N*_0_ as a function of *d*_*f*_ in Figure 2. The data support the theoretical result as the values can be fit extremely well by eq 21 throughout the entire range of *d*_*f*_. On the other hand, the data *cannot* be fit by logarithmic functional forms, which correspond to polynomial or power-law dependencies that are commonly associated with scaling arguments.^59^ Note that the statistical uncertainties in Figure 2 are smaller than the data points and therefore cannot explain the differences in the quality of fit. A detailed statistical analysis also offers strong support for the exponential form rather than the logarithmic (see Appendix B).

**Figure 3:**
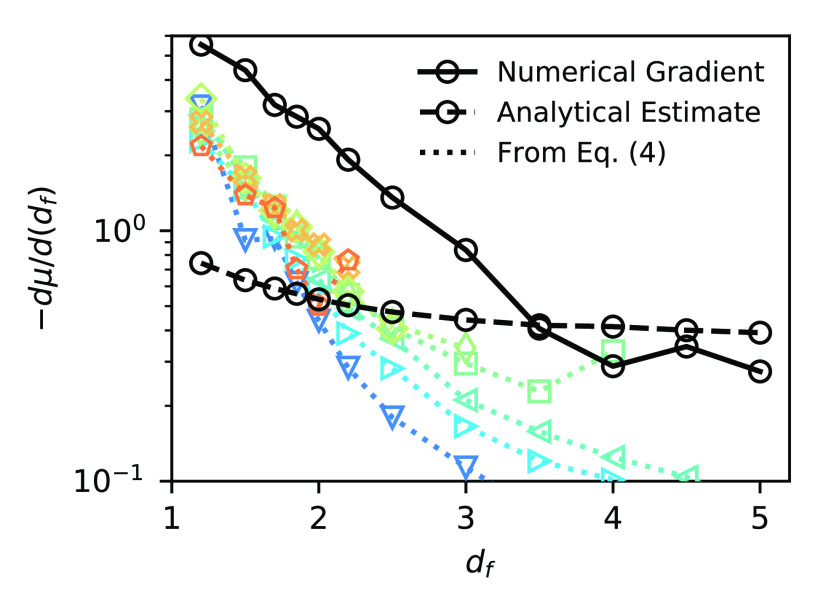
Derivatives dμ/d(*d*_*f*_) as calculated from numerical differentiation of the data in Figure 2 (black circles, solid lines), analytical estimate (black circles, dashed lines), and according to eq 8 (colored symbols, dotted lines). *N* increases as the color changes from blue to orange.

#### 3.2.4 Finite Size Effects

As mentioned above, the functions *f*_1_(*d*_*f*_) ≈ *f*_1_ and *f*_2_(*d*_*f*_) ≈ *f*_2_ are approximately constant for the systems considered here. Thus, we should observe the relations *c*_2_ = *f*_1_ and *c*_3_ = −*f*_2_/*f*_1_. However, the analytical calculations predict values of *c*_2_ ≈ −0.065 and *c*_3_ ≈ −2.35 while the fitted parameters are *c*_2_ = −1.138 and *c*_3_ = 3.703. The source of this discrepancy is related to finite-size effects. Equation 9 agrees only semiquantitatively with the simulation data and tends to underestimate the mode amplitudes at small *q*, which in turn leads to underestimates for dμ/d(*d*_*f*_) (see the Supporting Information). As *N* is increased, these deviations decrease, although there is a fairly broad crossover between the two scaling regimes that is not captured by eq 9.

These finite size effects should be captured by eq 8, which applies for all *N*, not only the limit *N* → *∞*. To see this, we evaluate these derivatives from the mode amplitudes and compare with both the analytical estimates and a simple numerical differentiation. We see that as *N* increases, the derivatives approach the “true” values obtained from the data in Figure 2, demonstrating the validity of eq 8 and highlighting the importance of these corrections.

#### 3.2.5 Lower Dimensional Systems

**Figure 4:**
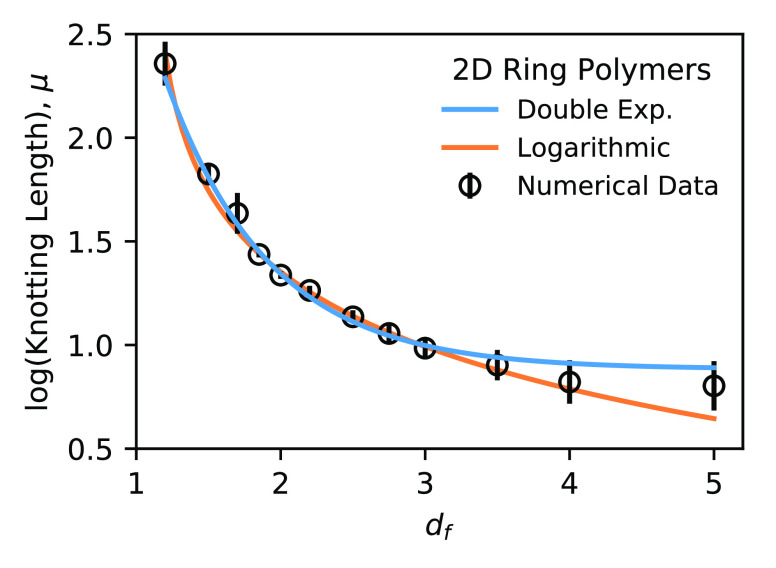
Logarithm of the knotting length ln *N*_0_ ≡ μ as a function of *d*_*f*_ for two-dimensional systems where knotting is defined by segment intersections. The double-exponential form eq 21 is valid in this lower dimensionality as well.

Interestingly, the general forms of our results do not depend on the dimensionality. Of course, knotted curves cannot exist in four dimensions,^35,62^ so only lower-dimensional analogues are relevant. In such circumstances, the exponential form of the “knotting” probability should still hold, and eqs 8–18 will remain unchanged except for modifications in the functions *f*_1_(*x*) and *f*_2_(*x*). To test this conjecture, we numerically examine the beta model in two dimensions, describing a given conformation as “knotted” if any two segments intersect; the resulting data are shown in Figure 4. We find that our results are perfectly applicable to this scenario, as evidenced by the exponential character of μ as a function of *d*_*f*_. Note that power-law or linear relationships between *N*_0_ and *d*_*f*_ are once again unsatisfactory, as in the 3D case, with strong support from statistical analysis (see the Supporting Information).

### 3.3 Discussion

The key results of this paper are contained in eqs 4, 9, and 18 and represent a starting point for studying how topology and fractal dimension are coupled in ring polymer systems. Although we have discovered some fundamental phenomenology and identified the underlying physics, a number of questions, both old and new, remain unanswered. For example, our data suggest that as *d*_*f*_ → *∞*, *N*_0_ → 33. This value is certainly model-dependent but is much larger than the minimum stick number of six mentioned earlier. It is unclear if or how these two values are related to each other. At the other end of the spectrum, it is strange that the double-exponential form eq 21 holds for *d*_*f*_ < 1.7, for which we expect the Flory argument to break down; indeed, systems with small *d*_*f*_ still exhibit considerable swelling in the unknotted state (see Figure S1). Perhaps most importantly, we have not been able to formulate any simple physical reasoning for the double-exponential form of *N*_0_(*d*_*f*_). The complex, nontrivial dependence makes it challenging to compare systems with different *N* or *d*_*f*_ on equal footing (for example, by matching their segment densities or chain sizes). In turn, other topological properties such as knot complexity, knot type distributions, and linking probabilities are difficult to predict, although we expect similarly complicated functional forms.

It is also important to recognize the assumptions and limitations of the present calculation. We have assumed that an effective Hamiltonian may be defined for the *unconstrained* ensemble in which the Fourier modes are independent and Gaussian-distributed. Clearly this assumption is not without merit since it forms the basis of much of modern polymer theory.^59^ In fact, even some topologically constrained and self-avoiding systems satisfy one or both of these restrictions,^63,64^ whereas we only require it for the *unconstrained* model. Moreover, the covariances of the Fourier modes in ring polymers are identically zero.^65^ While this does not prove statistical independence, it does offer hope that any correlations may be neglected with relative safety. In writing eq 9, we have also assumed that self-avoiding fractal polymers of arbitrary *d*_*f*_ are also self-similar; that is, they have a well-defined fractal dimension. Fortunately, this assumption has some support in the literature.^31^ Importantly, the results place no restrictions on the values of χ or γ, so the form of eq 12 is universal for all systems with a two-fractal character and a specified crossover length scale. It is perfectly natural that the result does not depend strongly on molecular details because the small length scale properties are unaffected by topological restrictions and therefore do not contribute to changes in *N*_0_. Indeed, the only model-dependent part of our results is a factor of ln(π/2), which appears in the function *f*_2_(*x*). Changes in the algebraic functions, however, do not alter the exponential character of μ or the resulting double-exponential character of *N*_0_, which follows from eq 12.

However, one must be cautious in interpreting the results: in this work, we have examined the effect of fractal dimension on *N*_0_ with all other considerations equal. In other words, we vary fractal dimension within a single, particular polymer model, leaving other confounding factors untouched. By contrast, if one compares different systems/models in which both *d*_*f*_ and many other parameters are varied, such a comparison loses its usefulness. For instance, models of polymer globules (either fractal or equilibrated) involve considerations of local bead packing and fluid structure, which are entirely absent in idealized random walks. Meanwhile, local fluid structure takes on an entirely different character in commonly used lattice models. Such variations in local conformation greatly modify the persistence lengths of the systems and frustrate any comparison on the basis of fractal dimension. Therefore, this kind of analysis is not appropriate. To reiterate, the particular choice of model is unimportant so long as a well-defined fractal dimension exists and can be varied without significantly altering local chain conformations. This is not merely an academic exercise: the beta model (and other similar ones) were developed to help understand the structure and dynamics of chromosomal DNA,^21^ which is presently one of the most active areas of polymer and soft matter research.

## 4 Conclusion

The interplay between topology and fractal dimension is a relatively new field of study but has important physical implications. In particular, because the knotting probability is intimately related to topological contributions to the free energy, we anticipate that our results will be helpful in understanding a variety of (bio)polymer systems. For example, in concentrated ring polymer solutions and melts, the polymer conformations are subject to topological constraints that prevent both knotting and linking. Similarly, the unknottedness of chromatin is believed to be crucial for its biological function,^15^ and the mechanics of other DNA-based systems such as Olympic gels^66^ and kinetoplasts^67^ will depend strongly on the presence of knotted moieties.^68−70^ Many of these systems also exhibit nontrivial self-similarity, making these results—and this subject more broadly—an exciting area of inquiry.

## Appendix A. Knotting between Blobs

In the blob picture discussed in the main text, knots were assumed to form within blobs or at the whole-chain level. It is also conceivable that knots form due to interactions between blob surfaces which would be lost upon further coarse-graining. Here we consider the effect of these interactions at the scaling level. We begin by modifying eq 5 of the main text to include the contributions of blob–blob interactions to the knotting probability:

22

The new third term on the right represents the probability that the ring is knotted at the interblob level and *not* at the intrablob or whole-chain scales (which are captured in the first two terms). The term ψ(*g*) is the probability that two contacting blobs are linked to one another (although we use the term “linked” rather loosely), and *M* is the number of such contacts. We estimate the number of contacting blobs at the mean-field level: *M* ≈ (*N*/*g*)^2^/*V* where  is the volume occupied by the polymer. We now substitute this estimate and rearrange eq 22:

23

As *N* → *∞*, the exponent  on the left approaches zero for *d*_*f*_ < 3 and unity for *d*_*f*_ = 3. In either case, the *N* dependence drops out, and the exponential form of the knotting probability is preserved. For larger fractal dimensions, this term actually increases with *N*, so the dependence cannot be ignored on the same grounds. However, for such compact polymers, the blobs are highly overlapping, so we expect that most of the knotting associated with blob–blob interactions should be accounted for in the whole-chain-scale knotting, i.e., the first term in eq 22, leaving blob surface interactions as a small correction.

## Appendix B. Statistical Analysis

Here we detail the statistical analysis used to produce error estimates in Figure 1 and to verify the superior performance of the double-exponential form in Figures 2 and 4. Each simulation with a given set of parameters {*N*, *d*_*f*_} generates a series of independent configurations which are either knotted or unknotted according to the probability *P*_0_(*N*, *d*_*f*_). Formally, this may be considered a Bernoulli process. To estimate the uncertainty in the observed value of *P*_0_, we use the fact that in Bayesian inference the conjugate prior distribution of a Bernoulli process is the beta distribution, whose parameters are determined by the number of positive (unknotted) and negative (knotted) observations.^71^ This models the distribution of the *true* probability given the observed outcomes. Because we conduct many trials for each {*N*, *d*_*f*_}, the distributions become approximately Gaussian, so we estimate the uncertainties in Figure 1 as double the variances of the associated beta distributions.

To determine the uncertainty in the values of μ ≡ ln(*N*_0_), we use a direct sampling method. From each of the beta distributions determined above, we draw 10^4^ random samples, fitting each set to eq 4 to determine *N*_0_(*d*_*f*_). We find that the resulting distributions of μ are approximately normal and therefore use the associated variances in fitting the data of Figures 2 and 4. The resulting 95% confidence intervals are smaller than the data markers in Figure 2 and are therefore omitted in that graph.

To compare the exponential and logarithmic fits of the data, we use the Bayesian information criterion (BIC), which is commonly used in model comparison and selection.^72^ Roughly speaking, the BIC reflects the likelihood of observing the data given the optimized model, with lower values corresponding to more likely models. Models with a greater number of parameters are also penalized, though that is not a concern for the present comparison. Note that the absolute BIC values are not important, only the differences between those of the models under consideration. For our purposes, noting that the distributions of μ are approximately normal and similar in variance, the BIC can be approximated (up to an additive constant) as

24

where *n* is the number of data points (12) and RSS is the residual sum of squares. As a general guideline,^72^ differences of two or more cannot be explained by random fluctuations in the data and constitute meaningful evidence that one model is better than another. Differences of 10 or more essentially eliminate the inferior model from consideration. In our case, we observe BIC’s roughly 22.4 and 5.8 points lower for the exponential fits compared to the logarithmic fits in three and two dimensions, respectively. As a result, we may say that the data strongly favor the exponential fit rather than the logarithmic, demonstrating that typical scaling forms are not appropriate for describing the dependence of random knotting length on the fractal dimension.

## Supporting Information Available

Additional figures concerning the size and structure of knotted and unknotted rings of various fractal dimensions as well as a graph of the algebraic functions, eqs 19 and 20 (PDF)

## Footnotes

a In earlier literature, the exponent χ in the eigenvalues λ_*q*_^(χ)^ is denoted instead by β. However, we have used a different symbol to avoid confusion with the inverse temperature, also commonly denoted β.

b The blobs are effectively linear (open) chains, for which knots do not formally exist. Therefore, knotting can only be defined after some chain closure procedure which joins the two free ends. Accordingly, we assume that the function ϕ(*g*) describes the probability that the associated linear chain is knotted after this (unspecified) procedure.
