# Supplementary material for: Random Knotting in Fractal Ring Polymers
Source: Macromolecules. 2022 Sep 8;55(18):8409–17. doi: 10.1021/acs.macromol.2c01676 (PMC9520986; doi:10.1021/acs.macromol.2c01676)
Supplement: Supplementary file 1 — ma2c01676_si_001.pdf [file ma2c01676_si_001.pdf]

Supporting Information: Random Knotting in Fractal Ring Polymers

Phillip M. Rauscher*,† and Juan J. de Pablo*,†,‡

†*Pritzker School of Molecular Engineering, University of Chicago, Chicago, Illinois 60637, USA*

‡*Materials Science Division (MSD) and Center for Molecular Engineering (CME), Argonne National Laboratory, Lemont, Illinois 60439, USA*

E-mail: phillip.rauscher@solvay.com; depablo@uchicago.edu

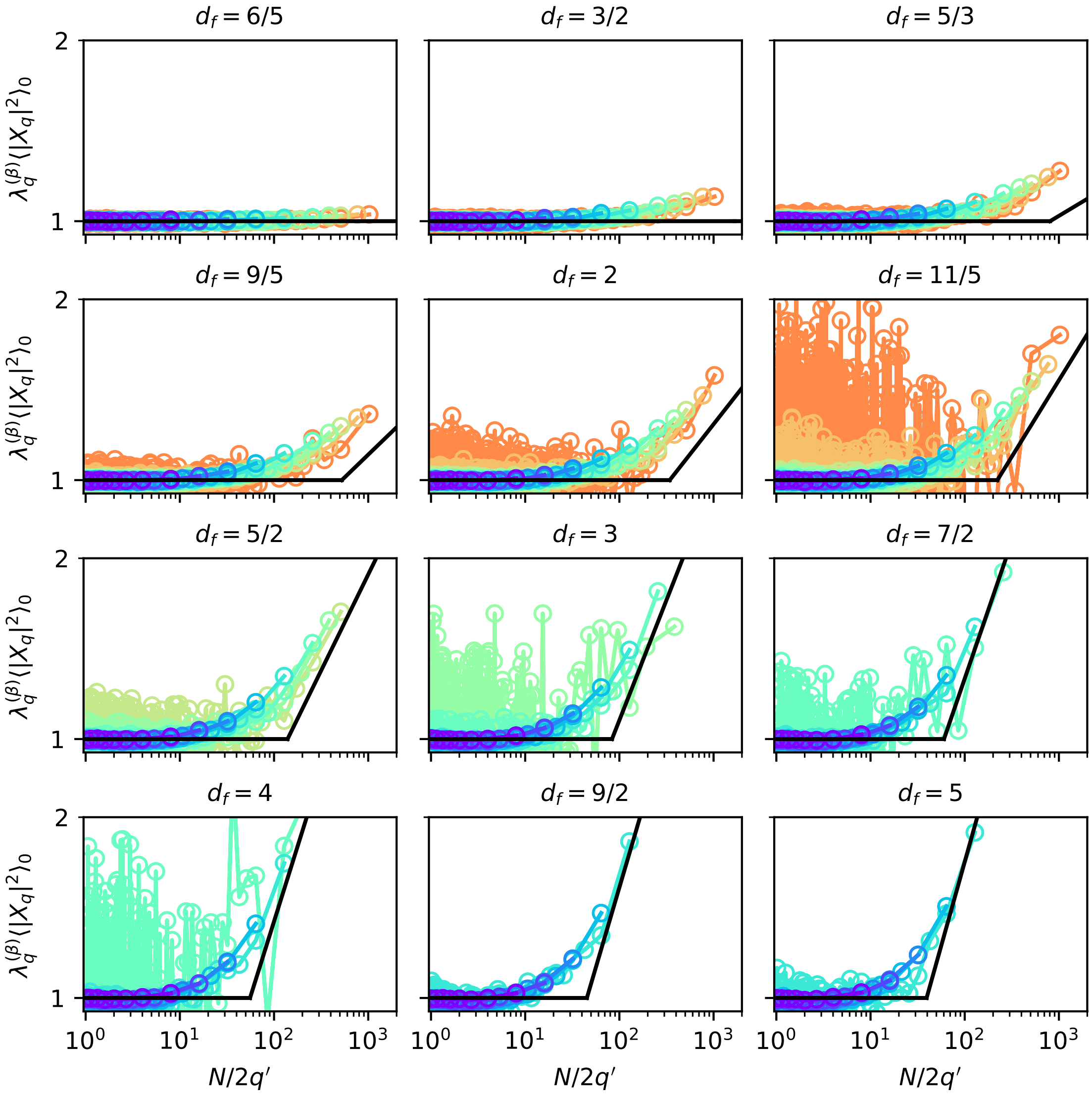

Figure S1: Normalized mean-squared mode amplitudes for unknotted ring polymers as a function of the segmental length scale $N/2q'$ where $q' = \min(q, N-q)$. Each color corresponds to a different value of N. The black lines show the predictions of Eq. (9). Note that both axes are logarithmic.

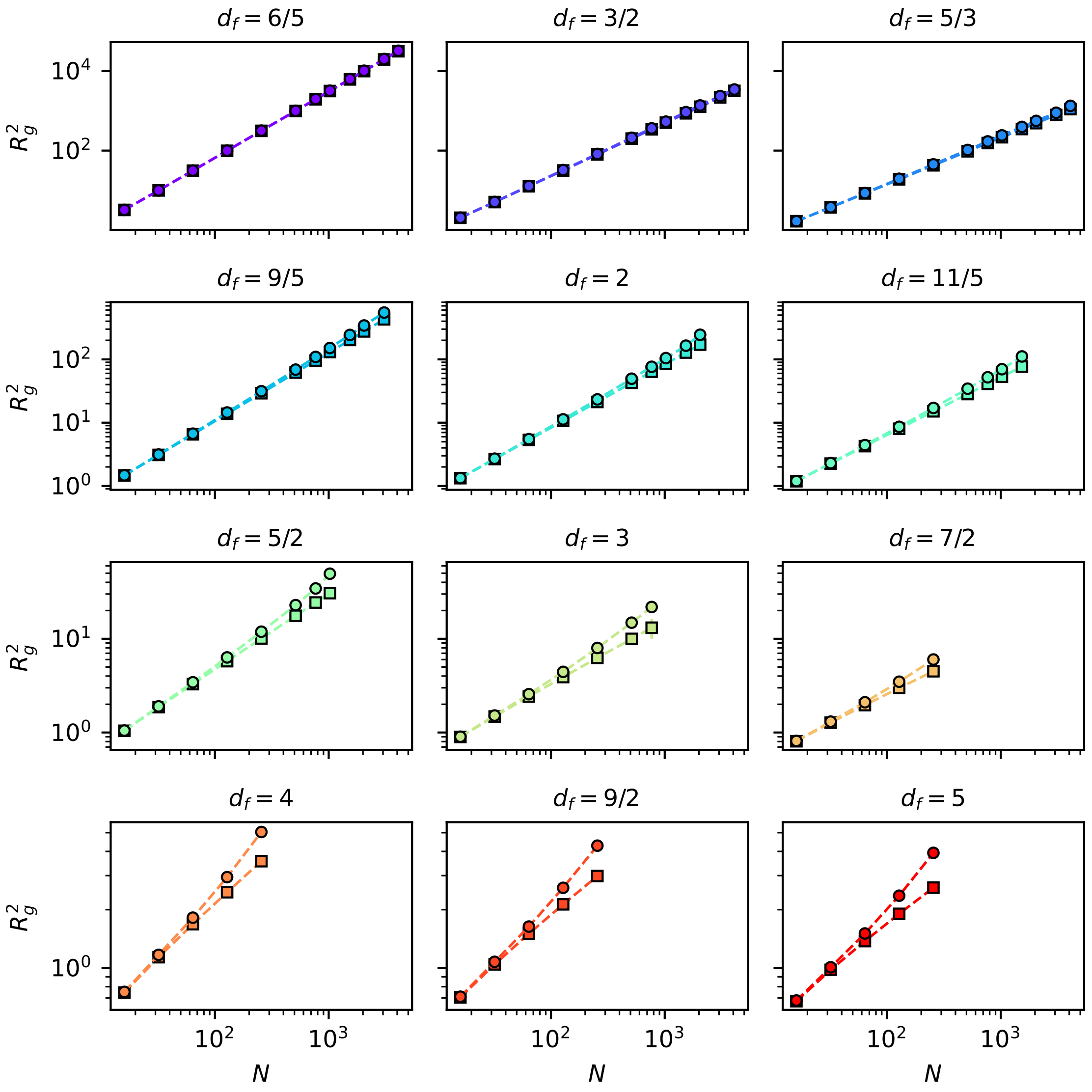

Figure S2: Mean squared radius of gyration for unknotted (circles) and ideal (squares) ring polymers as a function of the degree of polymerization N. The values of d_f corresponding to the different colors are the same as those in Fig. 1. Error bars are smaller than the size of the data points.

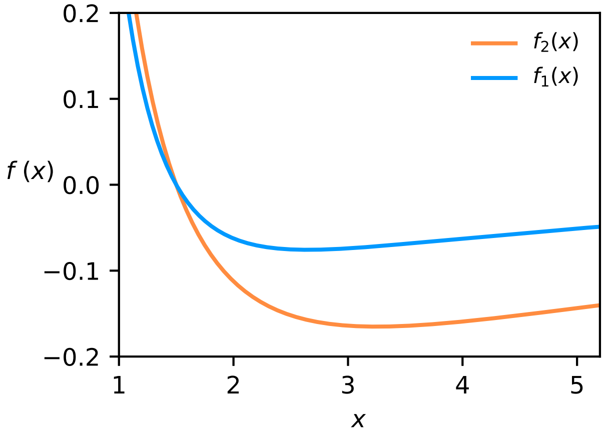

Figure S3: The algebraic functions defined in Eqs. (20).
